# Supplementary material for: PtWAVE: a high-sensitive deconvolution software of sequencing trace for the detection of large indels in genome editing
Source: BMC Bioinformatics. 2025 Apr 29;26:114. doi: 10.1186/s12859-025-06139-8 (PMC12039204; doi:10.1186/s12859-025-06139-8)
Supplement: Supplementary file 1 — Supplementary Material 1 [file 12859_2025_6139_MOESM1_ESM.docx]

**-- Supplemental Methods, Figures, Tables, and References --**

**PtWAVE: A High-Sensitive deconvolution software of sequencing trace for the Detection of Large Indels in Genome Editing**

Kazuki Nakamae^1,2,*^, Saya Ide^1,2^, Nagaki Ohnuki^1,2^, Yoshiko Nakagawa^2^, Keisuke Okuhara^2,3^, and Hidemasa Bono^1,3,*^

^1^ Genome Editing Innovation Center, Hiroshima University, Hiroshima 739-0046, Japan

^2^ PtBio Inc., Hiroshima 739-0046, Japan

^3^ Graduate School of Integrated Sciences for Life, Hiroshima University, Hiroshima 739-0046, Japan

* Correspondence should be addressed to H.B. [bonohu@hiroshima-u.ac.jp](mailto:bonohu@hiroshima-u.ac.jp). Correspondence may also be addressed to K.N. [kazu](mailto:kazuki-nakamae@hiroshima-u.ac.jp)ki-nakamae@hiroshima-u.ac.jp.

Kazuki Nakamae, Ph.D.

Genome Editing Innovation Center, Hiroshima University,

3-10-23 Kagamiyama, Higashi-Hiroshima, Hiroshima 739-0046, Japan

E-mail: [kazuki-nakamae@hiroshima-u.ac.jp](mailto:kazuki-nakamae@hiroshima-u.ac.jp)

Tel: +81-82-424-4013

Fax: +81-82-424-3990

Hidemasa Bono, Ph.D.

Graduate School of Integrated Sciences for Life, Hiroshima University,

3-10-23 Kagamiyama, Higashi-Hiroshima, Hiroshima 739-0046, Japan

E-mail: [bonohu@hiroshima-u.ac.jp](mailto:bonohu@hiroshima-u.ac.jp)

Tel.: +81-82-424-4013

**Supplemental Methods, Figures, Tables, and References**

**Contents**

1. Supplementary Methods. . . . . . . . . . . . . . . . . . . . . . . . . . . . . . . . . . . . . . . . . . . . . . . . . . . . .4
2. Supplementary Figures . . . . . . . . . . . . . . . . . . . . . . . . . . . . . . . . . . . . . . . . . . . . . . . . . . . . . .7
3. Supplementary Tables . . . . . . . . . . . . . . . . . . . . . . . . . . . . . . . . . . . . . . . . . . . . . . . . . . . . . . 22
4. Supplementary Sequence . . . . . . . . . . . . . . . . . . . . . . . . . . . . . . . . . . . . . . . . . . . . . . . . . . . 31
5. Supplementary References . . . . . . . . . . . . . . . . . . . . . . . . . . . . . . . . . . . . . . . . . . . . . . . . . . 32

**1 Supplementary Methods**

**Benchmarking and performance comparison using raw sequencing data from artificially mixed dsDNA**

Raw sequencing data for benchmarking was generated by *in vitro* experiments. The templates of Sanger sequencing were *in vitro* samples consisting of artificially synthesized 538 bp DNA, which imitated the wild-type sequence of Pear1 (chr3:87,685,636-87,686,173 (-)) on mouse genome assembly mm39 (named wild-type dsDNA), mixed with the artificially synthesized 453 bp DNA, which imitated the 85-bp deletion sequence of wild-type dsDNA (named large-deletion dsDNA), in a wide range of ratios (Fig. 3A-B; Supplementary Sequence S1-2). The WT DNA sequence has a SpCas9 target (5´-GGCGATCGAGTGTATCACGC NGG-3´), where our groups had previously confirmed that the 85 bp deletion in mouse blastocyst analysis using SpCas9-sgRNA ribonucleoprotein (unpublished data). The DNA sequencing template mixed to be a total of 0.12 pmol (normal conc.) or 0.012 pmol (low conc.), and the forward sequencing primer oligonucleotide (5´-GCTGAGGGTGATGGGTTTGA-3´) and reverse sequencing primer oligonucleotide (5´-CCACATGCTGAGGGTGAGAG-3´) was added 6.2 pmol in each mixture with tris buffer solution. The Sanger sequencings were performed with three biological replicates for every ratio (0, 5, 10, 20, 50, 80, 90, 95, 99, 100 percent of the large-deletion dsDNA), concentrations (normal conc. and low conc.), and primers (forward and reverse). The preparations of samples using different primers were performed on other days. The list of sequencing samples is shown in Table S2. Both wild-type dsDNA and large-deletion dsDNA were synthesized and delivered as dried DNA by Twist Bioscience. The Sanger sequencings were conducted by the FASMAC sequencing service.

The sequencing data was analyzed by PtWAVE and other TIDE analysis tools such as TIDE [1], ICE [2], and DECODR [3]. The analysis output was aggregated regarding editing efficiency and detection rate of 85bp large deletions. We compared the estimated and expected values and calculated the coefficient of determination (CoD) and coefficient of correlation (R) through a Python script. The CoD was calculated via the “metrics.r2_score” function of the scikit-learn module [4]. The R was calculated via the “stats.pearsonr” function of the SciPy module [5]. We used the CoD and the R to evaluate performance across different tools.

In the analysis using TIDE [1], the TIDE batch (<http://shinyapps.datacurators.nl/tide-batch/>) was employed. However, when indel predictions succeeded, the outputs, such as the editing efficiency and the fitting accuracy (R^2^), were unavailable for some sequencing trace data. For the data, analysis was conducted on the standard TIDE website (<http://shinyapps.datacurators.nl/tide/>), which is typically used for analyzing individual data. The editing efficiency and the fitting accuracy (R^2^) from the TIDE batch and standard TIDE website were used in the benchmarking analysis.

In the analysis using ICE (<https://ice.synthego.com/>) [2], results were obtained in batch analysis mode. The ICE scores were interpreted as the editing efficiency.

In the analysis using DECODR (<https://decodr.org/batch>) [3], the editing efficiency was determined by summing up all detected indels through homemade Python scripts.

In PtWAVE, the internal script-generated indel.json file was read to aggregate editing efficiency (editing_eff), the fitting accuracy (r_sq), and the Bayesian Information Criterion (BIC) through homemade Python scripts.

The used sequencing data and the used homemade script were deposited on the following GitHub repository (<https://github.com/KazukiNakamae/EditingSeq_Decomposition_HiroshimaUniv_PtBio_Benchmarking_Dataset>).

**2 Supplementary Figures**


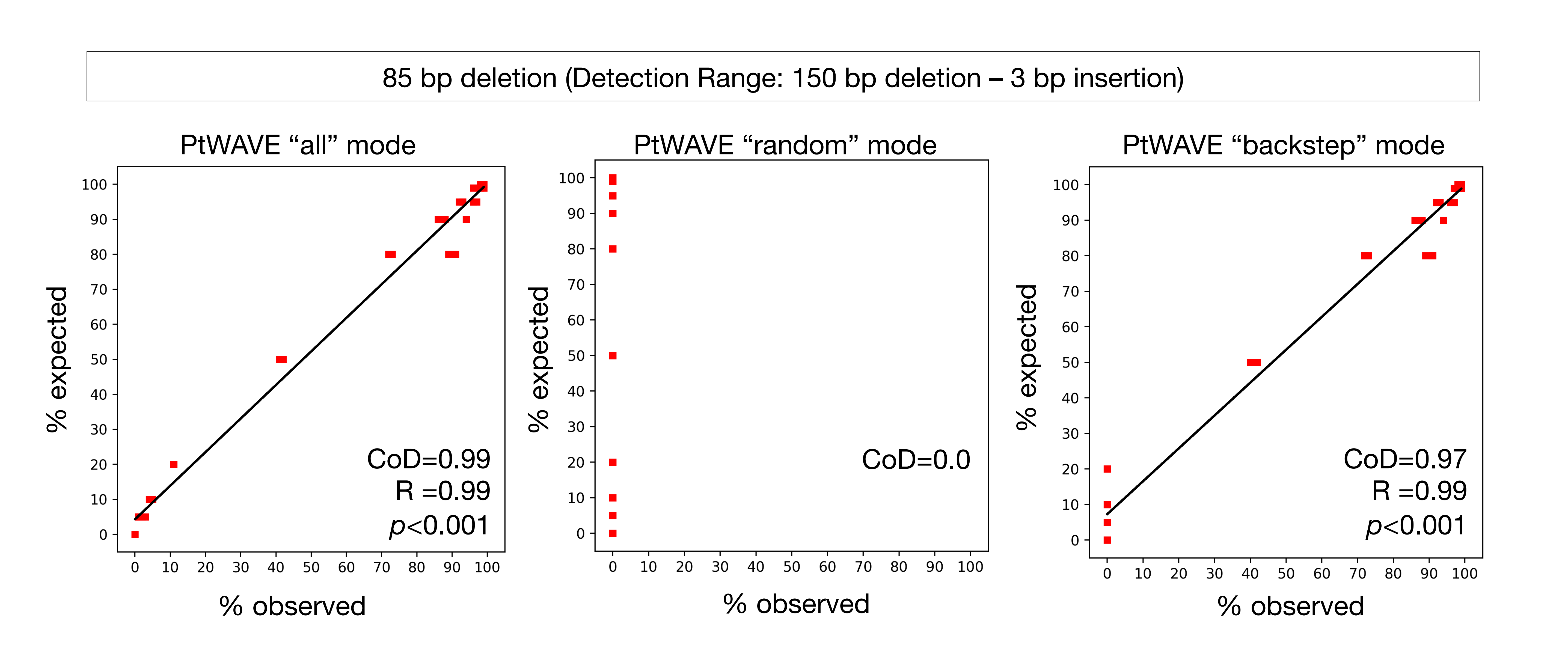


**Fig. S1: Evaluation of the large-deletion detection capability in various variable selection modes with the deletion detection range extended to 75 bp.**

The deletion detection range was extended to 75 bp, enabling the detection of a maximum of 150 bp. The detection rates of the 85 bp deletion dsDNA mixed at various ratios are plotted on the horizontal axis, with the initially expected detection rates on the vertical axis presented as a scatter plot. The approximation line was drawn using the linear_ model Linear Regression fit function from the scikit-learn module. The linear relationship was evaluated using the Coefficient of Determination (CoD), which has a maximum value of one and can take negative values. Correlations were assessed using Pearson's correlation coefficient (R), and the p-value from the no-correlation test was noted.


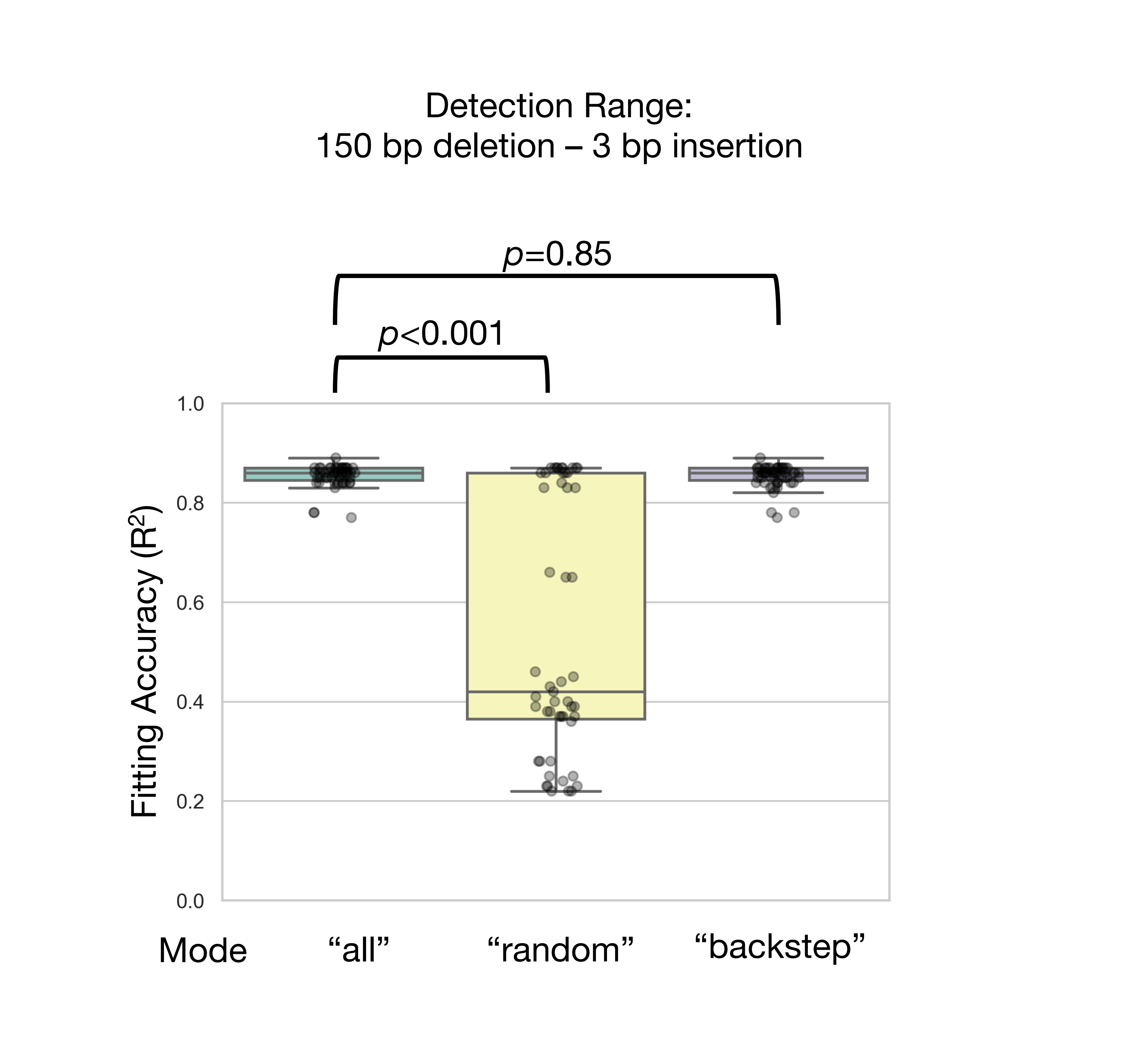


**Fig. S2: Evaluation of fitting accuracy in various variable selection modes with the deletion detection range extended to 75 bp.**

The fitting accuracies (R^2^) for each analysis are presented in Fig. S1 is depicted as a box plot. The horizontal axis represents the different variable selection modes and the vertical axis represents R^2^. The Wilcoxon signed-rank test was conducted as a two-sided test, and the p-value was noted. The Wilcoxon signed-rank test was performed using the stats.wilcoxon function from the SciPy module.


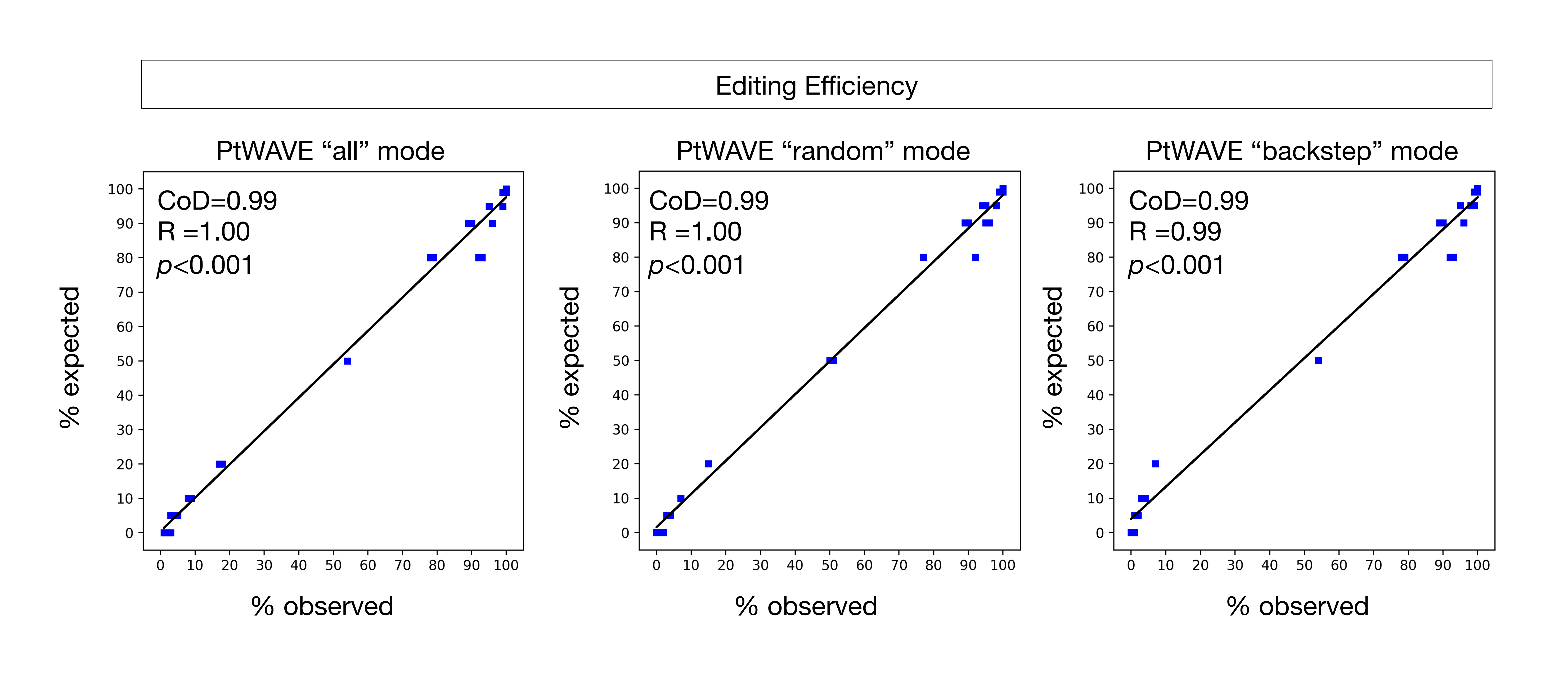


**Fig. S3: Evaluation of the editing efficiency estimation in various variable selection modes.**

The editing efficiencies at various ratios are plotted on the horizontal axis, with the initially expected editing efficiencies on the vertical axis presented as a scatter plot. The approximation line was drawn using the linear_ model Linear Regression fit function from the scikit-learn module. The linear relationship was evaluated using the Coefficient of Determination (CoD), which has a maximum value of one and can take negative values. Correlations were assessed using Pearson's correlation coefficient (R), and the p-value from the no-correlation test was noted.

**
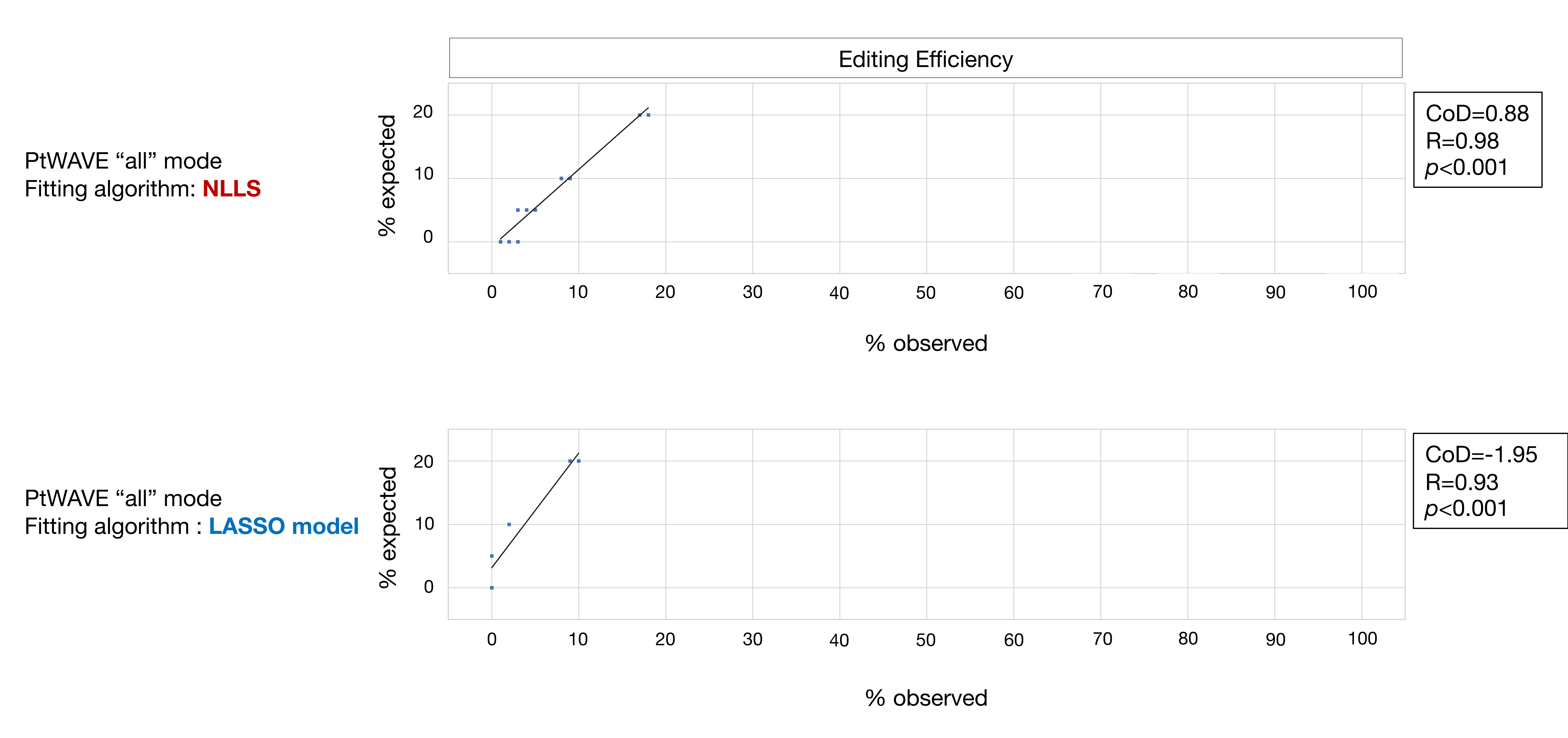
**

**Fig. S4: Evaluation of the editing efficiency estimation in “all” mode using various fitting algorithms using low-frequency samples.**

The editing efficiencies at various ratios are plotted on the horizontal axis, with the initially expected editing efficiencies of the low-frequency samples (0–20%) on the vertical axis presented as a scatter plot. The approximation line was drawn using the linear_ model Linear Regression fit function from the scikit-learn module. The linear relationship was evaluated using the Coefficient of Determination (CoD), which has a maximum value of one and can take negative values. Correlations were assessed using Pearson's correlation coefficient (R), and the p-value from the no-correlation test was noted.


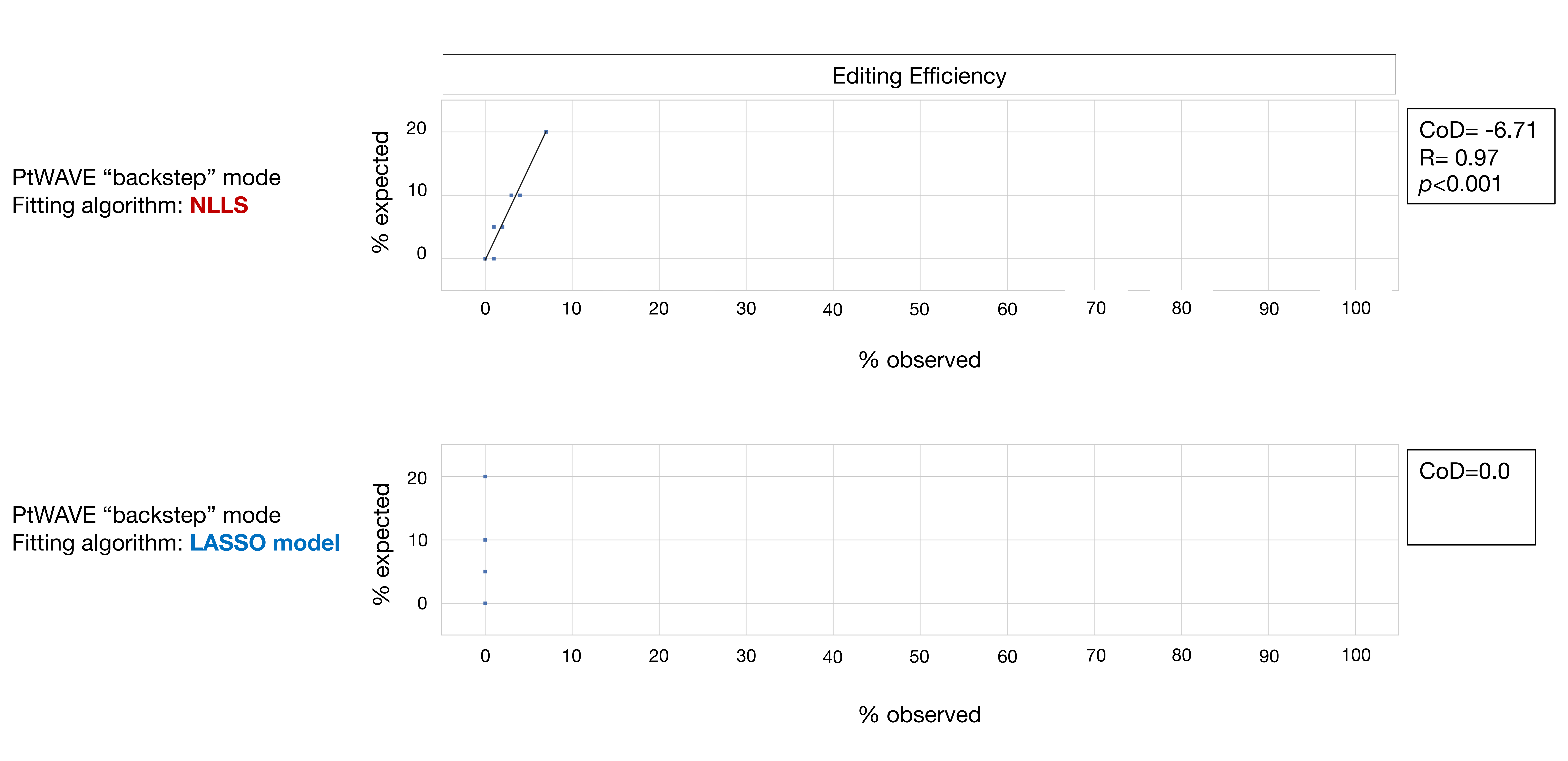


**Fig. S5: Evaluation of the editing efficiency estimation in “backstep” mode using various fitting algorithms using low-frequency samples.**

The editing efficiencies at various ratios are plotted on the horizontal axis, with the initially expected editing efficiencies of the low-frequency samples (0–20%) on the vertical axis presented as a scatter plot. The approximation line was drawn using the linear_ model Linear Regression fit function from the scikit-learn module. The linear relationship was evaluated using the Coefficient of Determination (CoD), which has a maximum value of one and can take negative values. Correlations were assessed using Pearson's correlation coefficient (R), and the p-value from the no-correlation test was noted.


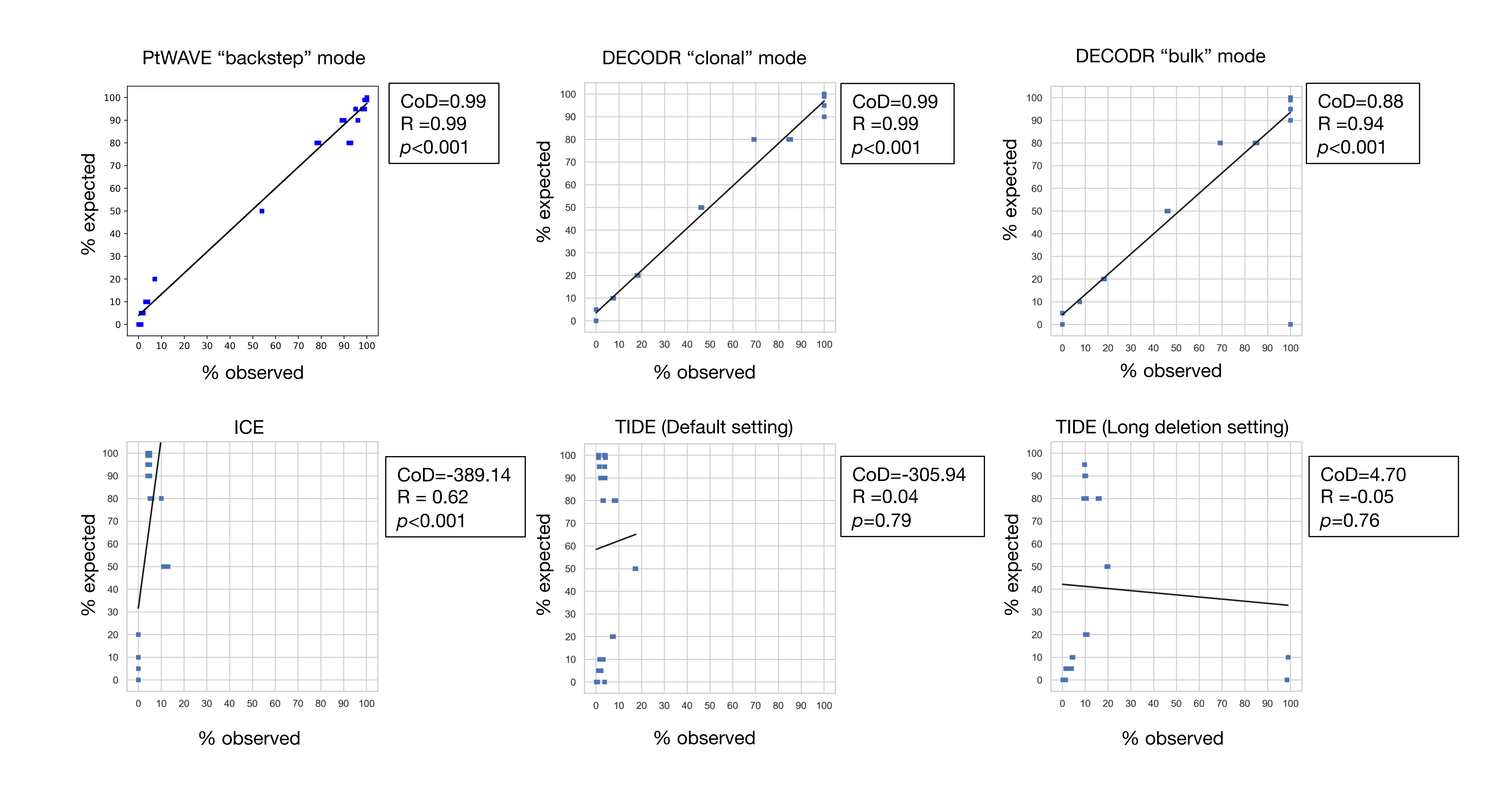


**Fig. S6: The evaluation of the editing efficiency estimation of various TIDE analysis tools.**

The editing efficiencies at various ratios are plotted on the horizontal axis. The initially expected editing efficiencies are plotted on the vertical axis. PtWAVE uses NNLS for modeling. The figure for PtWAVE is identical to Fig. S3. The default setting for TIDE is specified as "left boundary of alignment window=100, decomposition window: 115-685, indel size range=10," and the long deletion setting for TIDE is "left boundary of alignment window=1, decomposition window: 1-700, indel size range=50." A linear approximation curve was drawn using the linear_ model LinearRegression. fit function in the scikit-learn module. The linear relationship was evaluated using CoD, which can reach a maximum value of 1 and may take negative values. Correlations were assessed using R, and the p-value from the no-correlation test was noted. The absence of R and p-values indicated that the calculation was impossible.


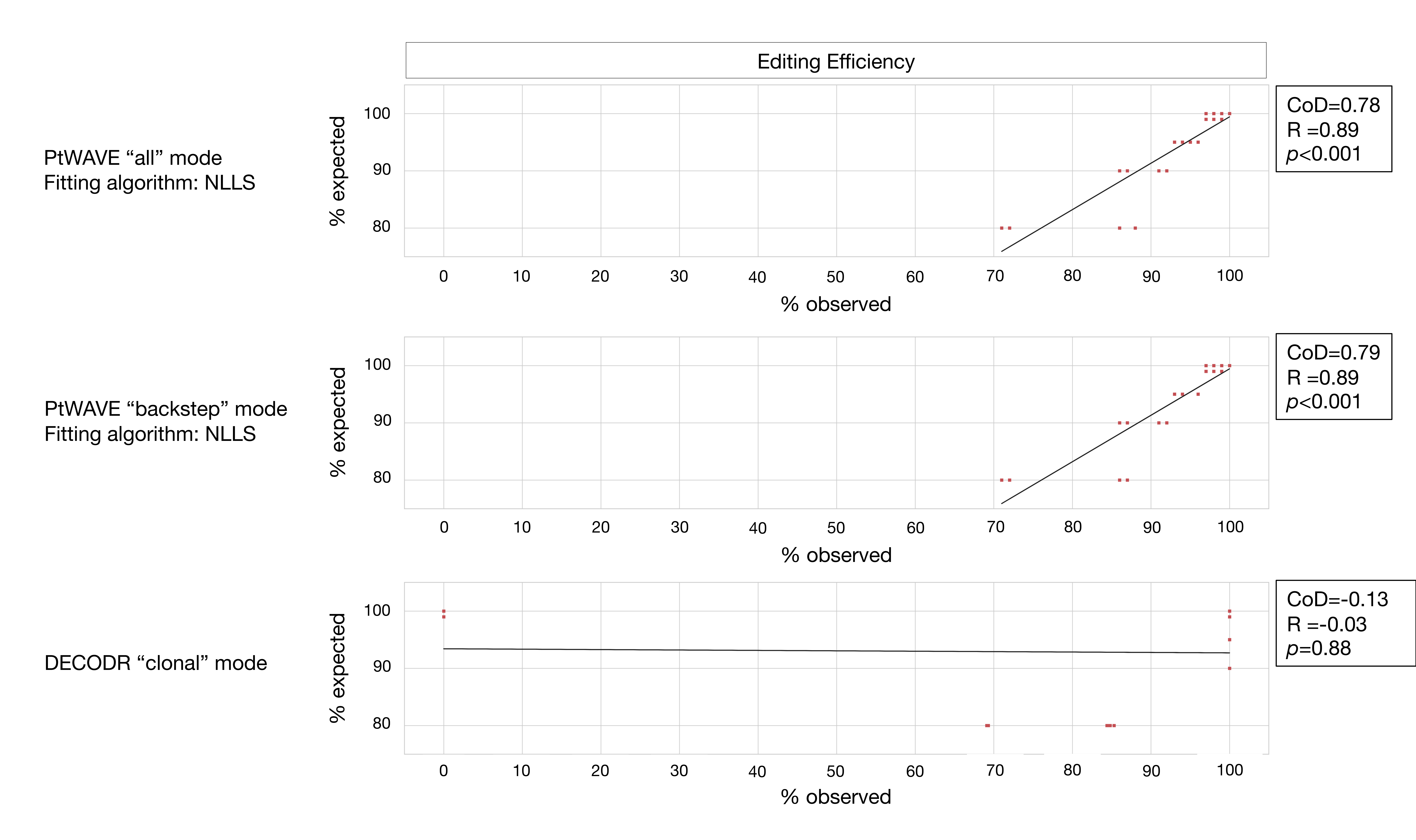


**Fig. S7: The evaluation of the large-deletion detection capability of PtWAVE and DECODR using high-frequency samples.**

The detection rates of the 85 bp deletion dsDNA mixed at various ratios are plotted on the horizontal axis. The initially expected detection rates of the high-frequency samples (80-100%) are plotted on the vertical axis. PtWAVE uses NNLS for modeling. A linear approximation curve was drawn using the linear_ model LinearRegression. fit function in the scikit-learn module. The linear relationship was evaluated using CoD, which can reach a maximum value of 1 and may take negative values. Correlations were assessed using R, and the p-value from the no-correlation test was noted. The absence of R and p-values indicated that the calculation was impossible.


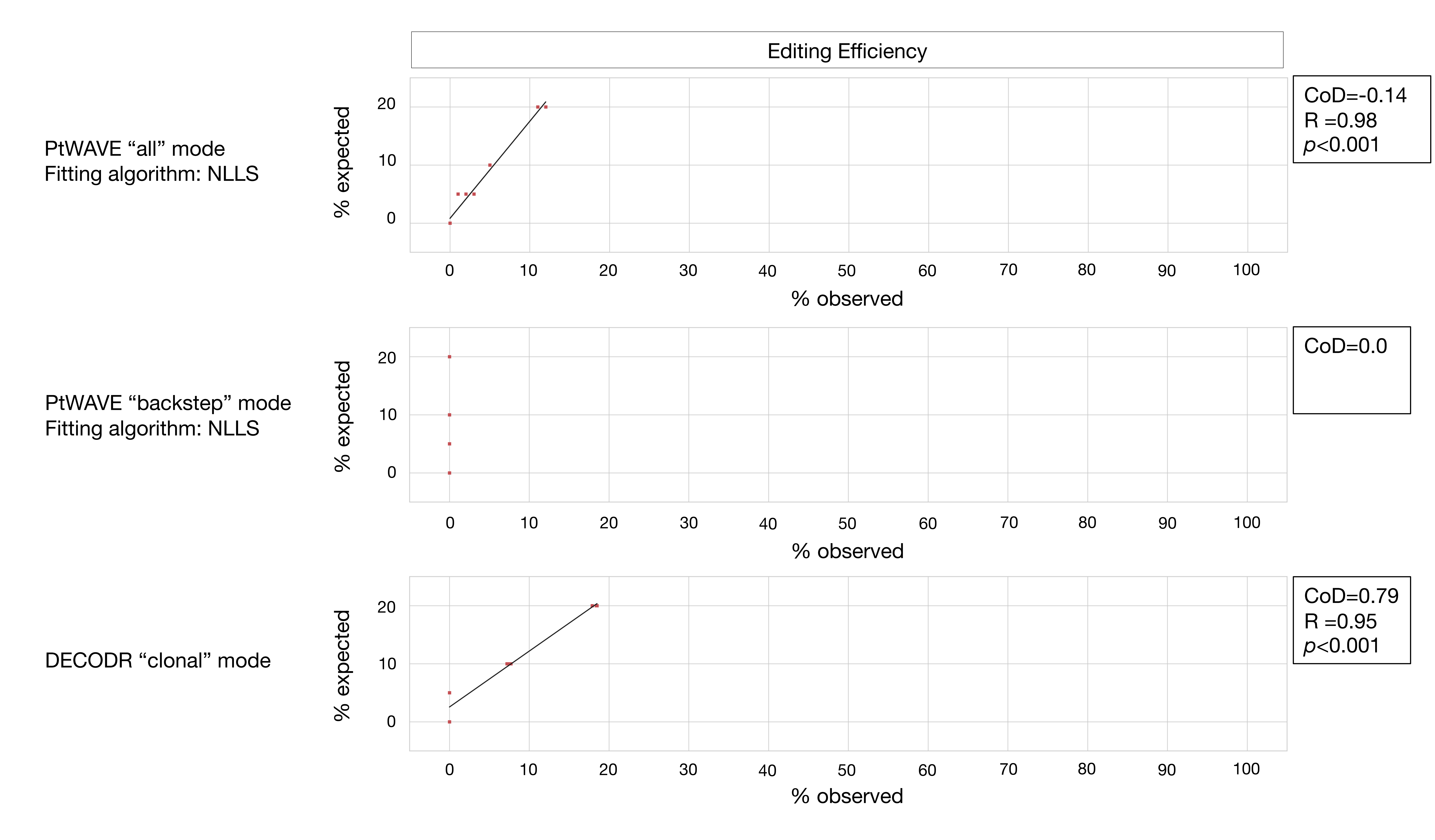


**Fig. S8: The evaluation of the large-deletion detection capability of PtWAVE and DECODR using low-frequency samples.**

The detection rates of the 85 bp deletion dsDNA mixed at various ratios are plotted on the horizontal axis. The initially expected detection rates of the low-frequency samples (0-20%) are plotted on the vertical axis. PtWAVE uses NNLS for modeling. A linear approximation curve was drawn using the linear_ model LinearRegression. fit function in the scikit-learn module. The linear relationship was evaluated using CoD, which can reach a maximum value of 1 and may take negative values. Correlations were assessed using R, and the p-value from the no-correlation test was noted. The absence of R and p-values indicated that the calculation was impossible.


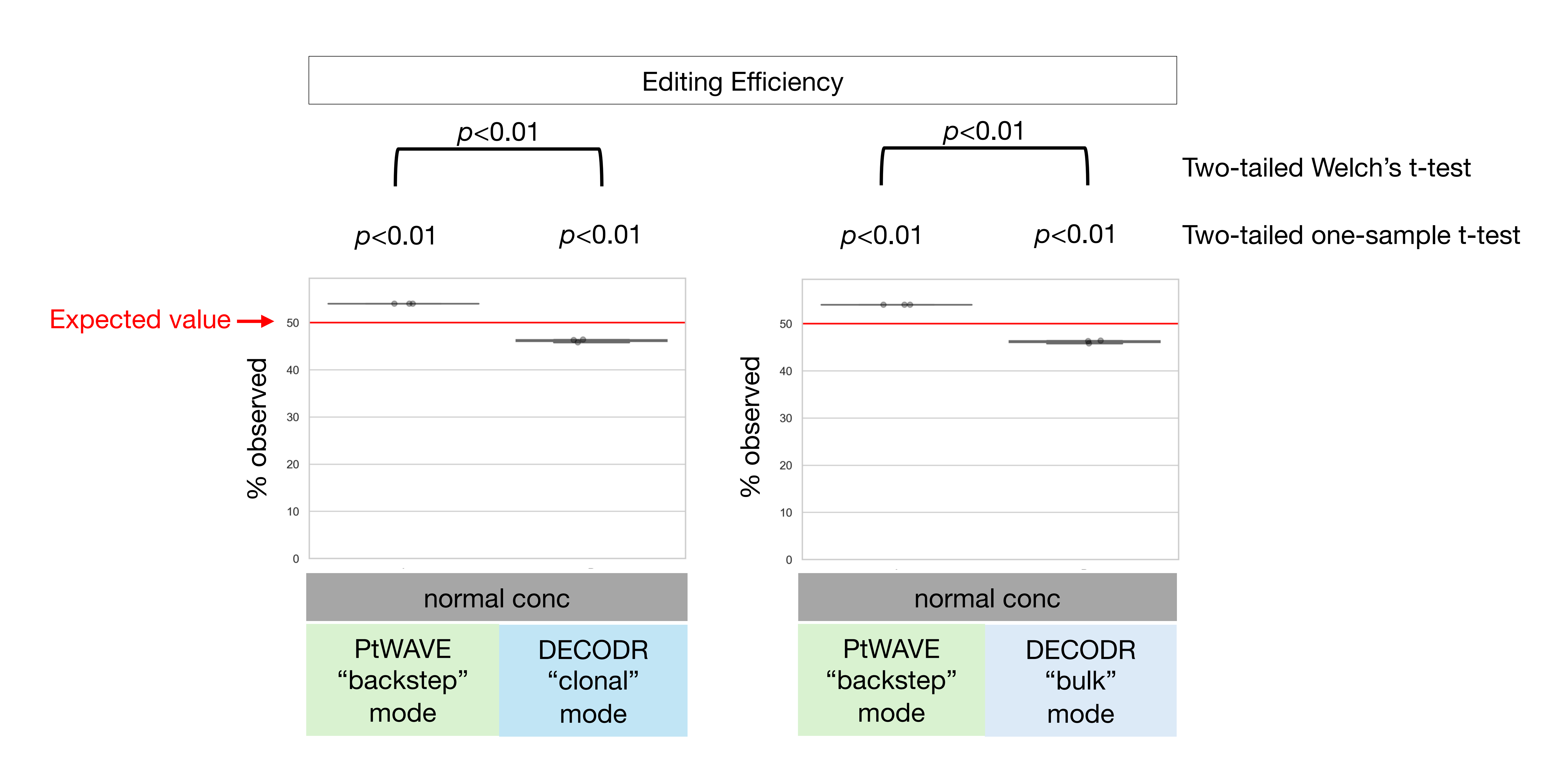


**Fig. S9: Comparison of the accuracy of editing efficiency estimation between PtWAVE and DECODR using specific frequency samples.**

The left box shows a comparison of the accuracy of the editing efficiency estimation between PtWAVE's backstep mode and DECODR's clonal DNA analysis mode as a box plot. The right box shows a comparison of the accuracy of the editing efficiency estimation between PtWAVE's backstep mode and DECODR's bulk DNA analysis mode as a box plot. The vertical axis represents the editing efficiency based on the tool results. All sequencing samples contained an equal mix of 85 bp deletion dsDNA and WT dsDNA. The expected editing efficiency was 50%, as indicated by the red lines. Two-tailed one-sample t-tests with a hypothesized mean value of 50 were conducted. A two-tailed Welch’s t-test was performed. The p-value of each t-test is noted above the box plots.


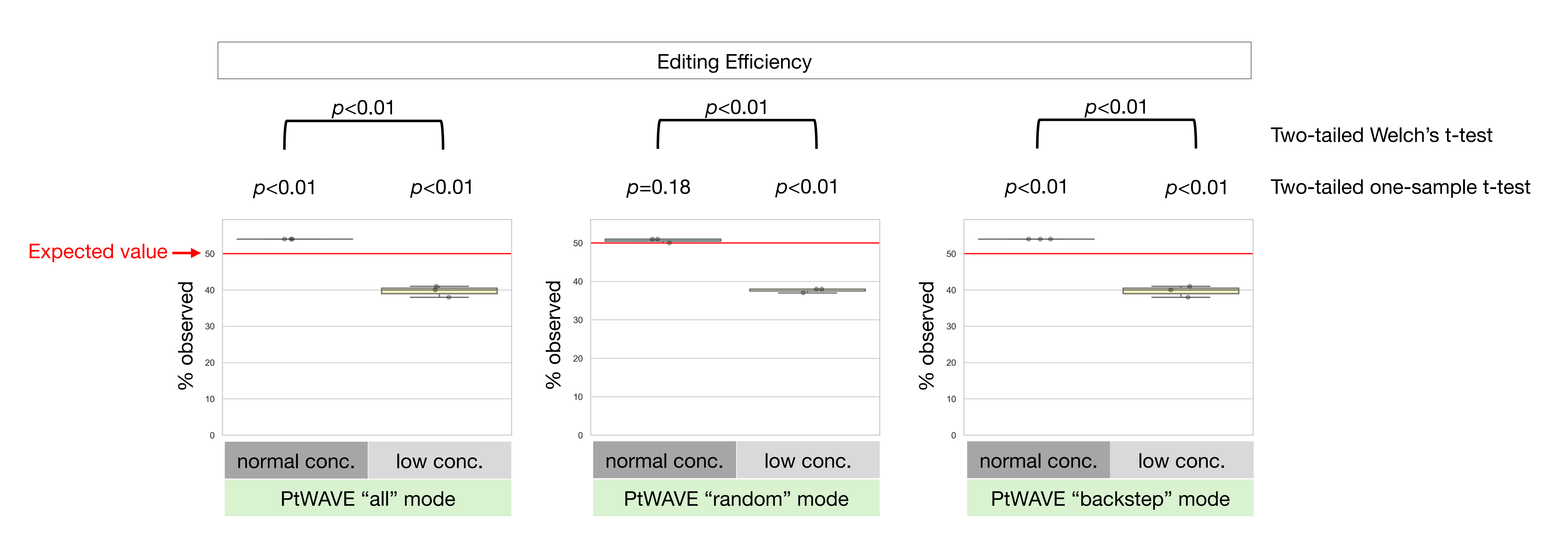


**Fig. S10: Comparison of the accuracy of editing efficiency estimation of PtWAVE in various variable selection modes between two concentrations using specific frequency samples.**

The left box showed the comparison of the accuracy of editing efficiency estimation of PtWAVE in “all” modes between two concentrations as two box plots. The center box showed the comparison of the accuracy of editing efficiency estimation of PtWAVE in “random” modes between two concentrations as two box plots. The right box showed the comparison of the accuracy of editing efficiency estimation of PtWAVE in “backstep” modes between two concentrations as two box plots. The vertical axis represents the editing efficiency based on the tool results. All sequencing samples contained an equal mix of 85 bp deletion dsDNA and WT dsDNA. The expected editing efficiency was 50%, as indicated by the red lines. Two-tailed one-sample t-tests with a hypothesized mean value of 50 were conducted. A two-tailed Welch’s t-test was performed. The p-value of each t-test is noted above the box plots.


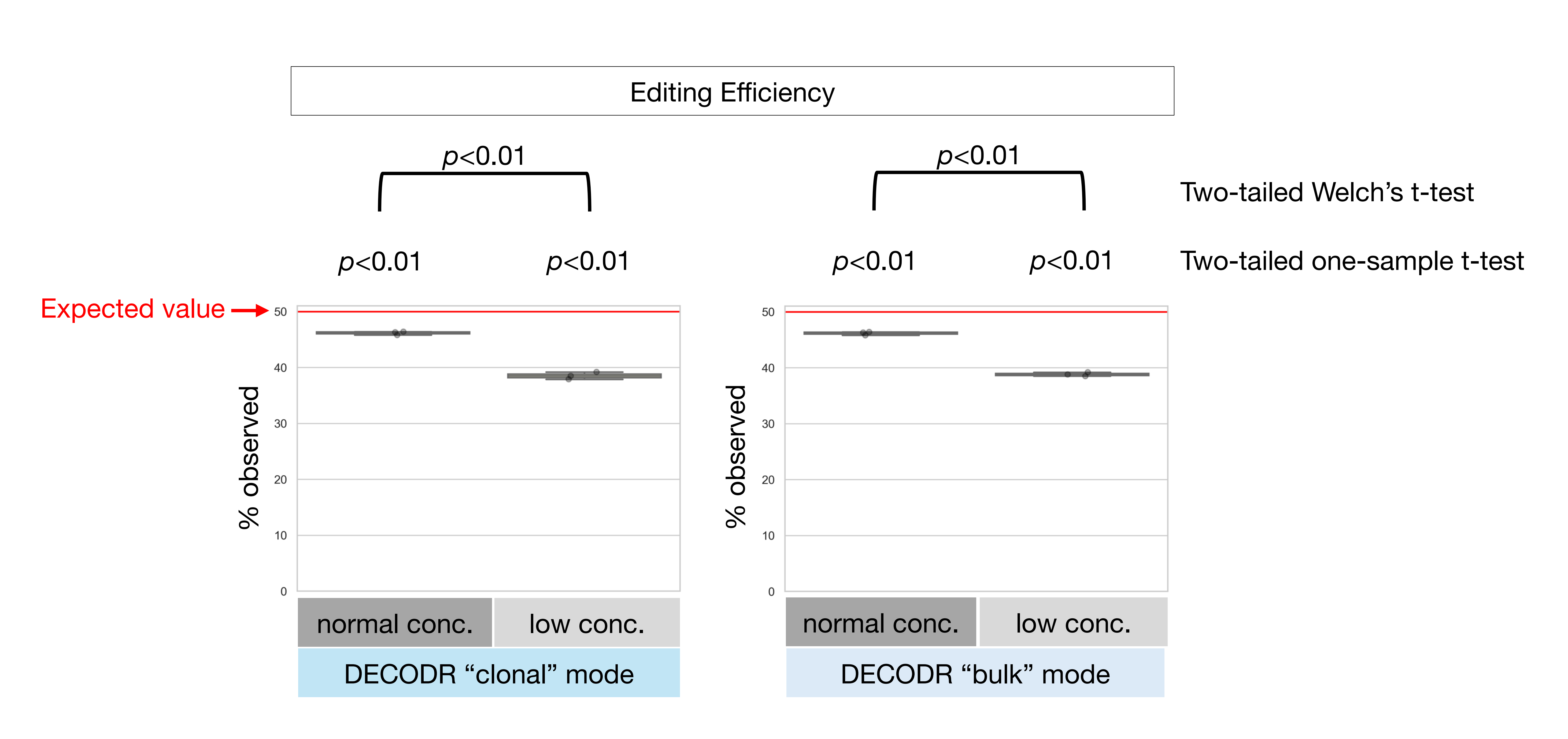


**Fig. S11: Comparison of the accuracy of editing efficiency estimation of DECODR in “clonal” and “bulk” modes between two concentrations using specific frequency samples.**

The left box showed the comparison of the accuracy of editing efficiency estimation of DECODR in “clonal” modes between two concentrations as two box plots. The right box showed the comparison of the accuracy of editing efficiency estimation of DECODR in “bulk” modes between two concentrations as two box plots. The vertical axis represents the editing efficiency based on the tool results. All sequencing samples contained an equal mix of 85 bp deletion dsDNA and WT dsDNA. The expected editing efficiency was 50%, as indicated by the red lines. Two-tailed one-sample t-tests with a hypothesized mean value of 50 were conducted. A two-tailed Welch’s t-test was performed. The p-value of each t-test is noted above the box plots.


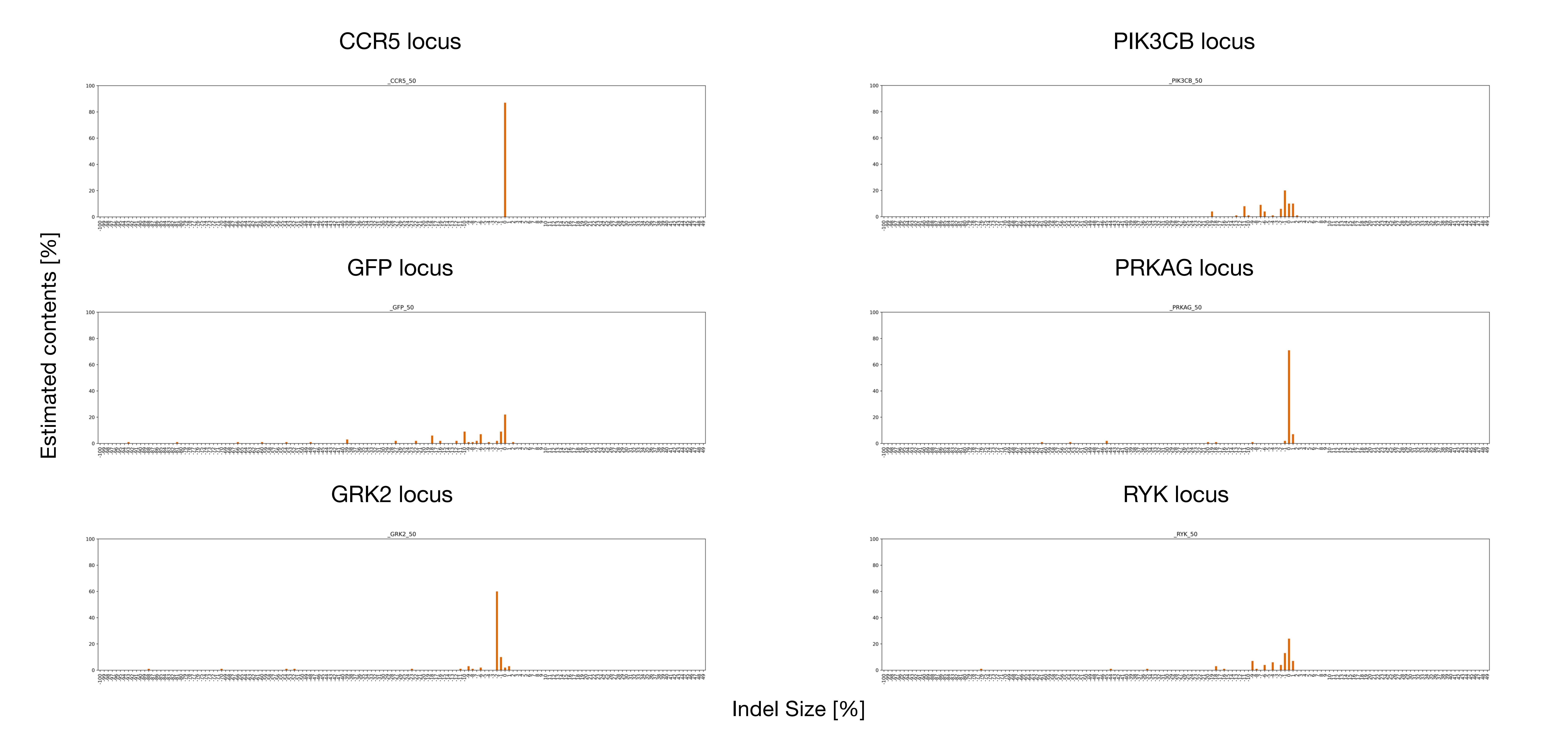


**Fig. S12: The indel distribution of six loci genome editing samples estimated by PtWAVE's backstep mode.**

Names of the target loci are shown in the boxes. The estimated indels from the 100 bp deletion to the 50 bp insertion are shown on the vertical axis in the indel distribution plot. The estimated contents of a specified indel size are shown on the vertical axis in the plot of the indel distribution. The detection range was set to the default setting, enabling the detection range to go from a 100 bp deletion to a 3 bp. The input sequencing files for CCR5 locus (5´-GTGAGTAGAGCGGAGGCAGG-3´) are “Hek CCR5 cas9 39FG14.ab1” and “Hek CCR5 UT 39FG13.ab1” [41]. The input sequencing files for PIK3CB locus (5´-TTGTTTGAACAGTTCCCAT-3´) are “19bp_guide_PIK3CB_experiment.ab1” and “19bp_guide_PIK3CB_control.ab1” [25, 40]. The input sequencing files for GFP locus (5´-GTGGTCACGAGGGTGGGCCA-3´) are “U2OS GFP cas9 vs GFP 39FF51.ab1” and “U2OS GFP UT ff4639FF50.ab1 [41]. The input sequencing files for PRKAG locus (5´-GGAAGTATACACGCTATTGT-3´) are “low_edit_PRKAG1_experiment.ab1” and “low_edit_PRKAG1_control.ab1” [25, 40]. The input sequencing files for GRK2 locus (5´- TGTATGAGTCGAAGATCTCC-3´) are “high_edit_GRK2_experiment.ab1” and “high_edit_GRK2_control.ab1” [25, 40]. The input sequencing files for RYK locus (5´- ATGTAAGGCAATATCACCAT-3´) are “low_ko_RYK_experiment.ab1” and “low_ko_RYK_control.ab1” [25, 40].


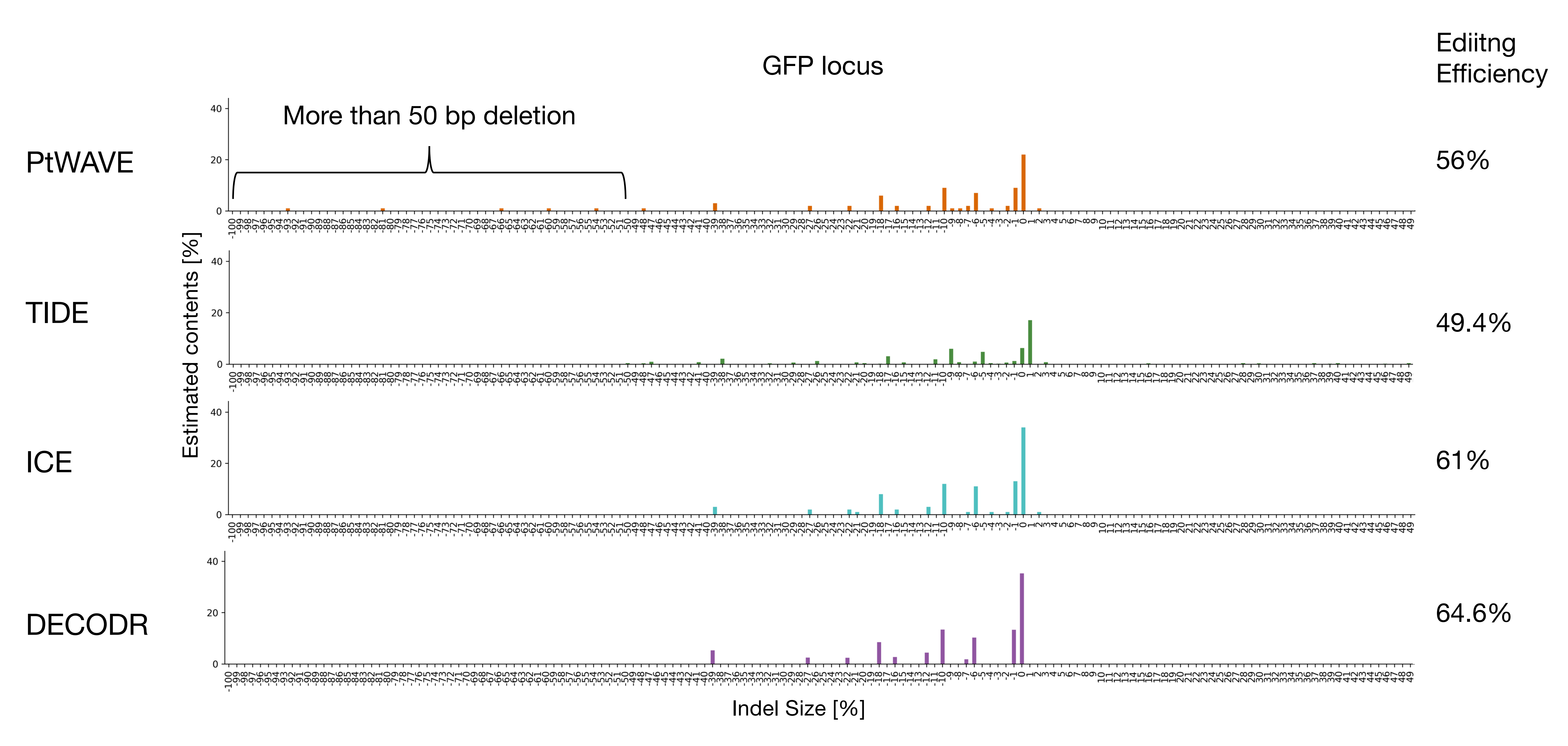


**Fig. S13: The indel distribution and ediitng efficiency of genome editing samples in the GFP locus estimated by TIDE analysis tools.**

The name of the TIDE analysis tool is indicated on the left side of the box. Editing efficiency, estimated using the TIDE analysis tool, is shown on the right side of each box. The estimated indels from the 100 bp deletion to the 50 bp insertion are shown on the vertical axis in the indel distribution plot. The estimated contents of a specified indel size are shown on the vertical axis in the plot of the indel distribution. The detection range of PtWAVE was set to the default settings, enabling a detection range from 100 bp to 3 bp. The brace in the box indicates a range of deletions greater than 50 bp. The input sequencing files for GFP locus (5´-GTGGTCACGAGGGTGGGCCA-3´) are “U2OS GFP cas9 vs GFP 39FF51.ab1” and “U2OS GFP UT ff4639FF50.ab1 [41].

**3 Supplementary Tables**

**Tab. S1**

| Python Modules | Version |
| --- | --- |
| altair | 5.2.0 |
| annotated-types | 0.6.0 |
| anyio | 4.2.0 |
| attrs | 23.2.0 |
| backports.zoneinfo | 0.2.1 |
| biopython | 1.78 |
| blinker | 1.7.0 |
| cachetools | 5.3.2 |
| certifi | 2023.11.17 |
| charset-normalizer | 3.3.2 |
| click | 8.1.7 |
| contourpy | 1.1.1 |
| cycler | 0.12.1 |
| exceptiongroup | 1.2.0 |
| fastapi | 0.100.0 |
| fonttools | 4.47.0 |
| gitdb | 4.0.11 |
| GitPython | 3.1.41 |
| h11 | 0.14.0 |
| idna | 3.6 |
| importlib-metadata | 6.11.0 |
| importlib-resources | 6.1.1 |
| Jinja2 | 3.1.3 |
| joblib | 1.3.2 |
| jsonschema | 4.20.0 |
| jsonschema-specifications | 2023.12.1 |
| kiwisolver | 1.4.5 |
| markdown-it-py | 3.0.0 |
| MarkupSafe | 2.1.3 |
| matplotlib | 3.7.1 |
| mdurl | 0.1.2 |
| numpy | 1.22.3 |
| packaging | 23.2 |
| pandas | 2.0.3 |
| Pillow | 9.5.0 |
| pip | 23.3.1 |
| pkgutil_resolve_name | 1.3.10 |
| protobuf | 4.25.2 |
| pyarrow | 14.0.2 |
| pydantic | 2.5.3 |
| pydantic_core | 2.14.6 |
| pydeck | 0.8.1b0 |
| Pygments | 2.17.2 |
| Pympler | 1.0.1 |
| pyparsing | 3.1.1 |
| python-dateutil | 2.8.2 |
| pytz | 2023.3.post1 |
| pytz-deprecation-shim | 0.1.0.post0 |
| referencing | 0.32.1 |
| requests | 2.31.0 |
| rich | 13.7.0 |
| rpds-py | 0.16.2 |
| scikit-learn | 1.0.2 |
| scipy | 1.7.3 |
| seaborn | 0.11.2 |
| setuptools | 68.2.2 |
| six | 1.16.0 |
| smmap | 5.0.1 |
| sniffio | 1.3.0 |
| starlette | 0.27.0 |
| streamlit | 1.24.1 |
| tenacity | 8.2.3 |
| threadpoolctl | 3.2.0 |
| toml | 0.10.2 |
| toolz | 0.12.0 |
| tornado | 6.4 |
| typing_extensions | 4.9.0 |
| tzdata | 2023.4 |
| tzlocal | 4.3.1 |
| urllib3 | 2.1.0 |
| uvicorn | 0.23.1 |
| validators | 0.22.0 |
| wheel | 0.41.2 |
| zipp | 3.17.0 |

**Tab. S1: Used Python Modules in PtWAVE.** The first column lists the names of Python modules used to run PtWAVE, while the second column shows the version information for each module

**Tab. S2**

| Sequencing Data [.ab1 file] | Large-deletion DNA [%] | Total Template DNA [pmol] | Replicate | Primer | Note |
| --- | --- | --- | --- | --- | --- |
| 1-1 | 0 | 0.12 | 1 | Forward |  |
| 1-2 | 0 | 0.12 | 2 | Forward |  |
| 1-3 | 0 | 0.12 | 3 | Forward |  |
| 2-1 | 5 | 0.12 | 1 | Forward |  |
| 2-2 | 5 | 0.12 | 2 | Forward |  |
| 2-3 | 5 | 0.12 | 3 | Forward |  |
| 3-1 | 10 | 0.12 | 1 | Forward |  |
| 3-2 | 10 | 0.12 | 2 | Forward |  |
| 3-3 | 10 | 0.12 | 3 | Forward |  |
| 4-1 | 20 | 0.12 | 1 | Forward |  |
| 4-2 | 20 | 0.12 | 2 | Forward |  |
| 4-3 | 20 | 0.12 | 3 | Forward |  |
| 5-1 | 50 | 0.12 | 1 | Forward |  |
| 5-2 | 50 | 0.12 | 2 | Forward |  |
| 5-3 | 50 | 0.12 | 3 | Forward |  |
| 6-1 | 80 | 0.12 | 1 | Forward |  |
| 6-2 | 80 | 0.12 | 2 | Forward |  |
| 6-3 | 80 | 0.12 | 3 | Forward |  |
| 7-1 | 90 | 0.12 | 1 | Forward |  |
| 7-2 | 90 | 0.12 | 2 | Forward |  |
| 7-3 | 90 | 0.12 | 3 | Forward |  |
| 8-1 | 95 | 0.12 | 1 | Forward |  |
| 8-2 | 95 | 0.12 | 2 | Forward |  |
| 8-3 | 95 | 0.12 | 3 | Forward |  |
| 9-1 | 100 | 0.12 | 1 | Forward |  |
| 9-2 | 100 | 0.12 | 2 | Forward |  |
| 9-3 | 100 | 0.12 | 3 | Forward |  |
| 10-1 | 0 | 0.12 | 1 | Forward | Control Sequence Data |
| 11-1 | 0 | 0.12 | 1 | Reverse |  |
| 11-2 | 0 | 0.12 | 2 | Reverse |  |
| 11-3 | 0 | 0.12 | 3 | Reverse |  |
| 12-1 | 5 | 0.12 | 1 | Reverse |  |
| 12-2 | 5 | 0.12 | 2 | Reverse |  |
| 12-3 | 5 | 0.12 | 3 | Reverse |  |
| 13-1 | 80 | 0.12 | 1 | Reverse |  |
| 13-2 | 80 | 0.12 | 2 | Reverse |  |
| 13-3 | 80 | 0.12 | 3 | Reverse |  |
| 14-1 | 90 | 0.12 | 1 | Reverse |  |
| 14-2 | 90 | 0.12 | 2 | Reverse |  |
| 14-3 | 90 | 0.12 | 3 | Reverse |  |
| 15-1 | 95 | 0.12 | 1 | Reverse |  |
| 15-2 | 95 | 0.12 | 2 | Reverse |  |
| 15-3 | 95 | 0.12 | 3 | Reverse |  |
| 16-1 | 100 | 0.12 | 1 | Reverse |  |
| 16-2 | 100 | 0.12 | 2 | Reverse |  |
| 16-3 | 100 | 0.12 | 3 | Reverse |  |
| 17-1 | 0 | 0.12 | 1 | Reverse | Control Sequence Data |
| 18-1 | 99 | 0.12 | 1 | Forward |  |
| 18-2 | 99 | 0.12 | 2 | Forward |  |
| 18-3 | 99 | 0.12 | 3 | Forward |  |
| 19-1 | 99 | 0.12 | 1 | Reverse |  |
| 19-2 | 99 | 0.12 | 2 | Reverse |  |
| 19-3 | 99 | 0.12 | 3 | Reverse |  |
| 20-1 | 0 | 0 | 1 | Forward | Primer Only |
| 20-2 | 0 | 0 | 2 | Forward | Primer Only |
| 20-3 | 0 | 0 | 3 | Forward | Primer Only |
| 21-1 | 0 | 0 | 1 | Reverse | Primer Only |
| 21-2 | 0 | 0 | 2 | Reverse | Primer Only |
| 21-3 | 0 | 0 | 3 | Reverse | Primer Only |
| 22-1 | 50 | 0.012 | 1 | Forward |  |
| 22-2 | 50 | 0.012 | 2 | Forward |  |
| 22-3 | 50 | 0.012 | 3 | Forward |  |
| 23-1 | 50 | 0.012 | 1 | Reverse |  |
| 23-2 | 50 | 0.012 | 2 | Reverse |  |
| 23-3 | 50 | 0.012 | 3 | Reverse |  |

**Tab. S2: Used sequencing data and its contents.** The first column specifies the file names of the used sequence data. The second, third, fourth, fifth, and sixth columns respectively represent the content of large-deletion DNA in each data, the total molar mass of the template DNA, the identification number of replicates, the sequencing primer, and other ancillary information. The sequence data is publicly available at the following GitHub repository: https://github.com/KazukiNakamae/EditingSeq_Decomposition_HiroshimaUniv_PtBio_Benchmarking_Dataset/tree/main/Dataset.

**[Tab.S3.xlsx]**

**Tab. S3: Summary of the benchmarking results for the dataset from in vitro artificially mixed dsDNA samples**

**[Tab.S4.xlsx]**

**Tab. S4: Summary of the benchmarking results for the published dataset**

**4 Supplementary Sequence**

**Sequence. S1: wild-type dsDNA**

GCTGAGGGTGATGGGTTTGATAATGTCATGCCCTCACTCTTAGCACCCCACCCGGCGAGTCCTCACTGGGTGTAACACTAGCCTTCTGGAGGCAATCCTAGAGTCGGGCAGCAATTATTCCCACTTTTCAGAGACGGAGCAGGAAAGAGCCTGGGAGTTTGAAGGGCACCATACAGTTCAGTCCGTCAGGGACCATCTGGGAGAGCCTGTAACCTTCCTGTCCCGAAACTCTCAGTTTGCTCTCCTCTCATCTCTCCCGTGGCGCAGGCGATCGAGTGTATCACGCAGGGCCGGGAGCTGGAGCGGCCGCGCGCCTGCCCTCCTGATGTCTACGCCATCATGCGAGGCTGCTGGCAGCGAGAACCGCAGCAACGCCTCAGCATGAAGGATGTGCACGCGCGTCTGCAGGCCCTGGCACAGGCGCCACCCAGTTACCTGGACGTTCTGGGCTAGGAGCCAAGTTTTGGTGCCAGGCCACCCTGGGCTCCCTCGGTGCCCAGGAGCTACCACATTCAAGTCTCTCACCCTCAGCATGTGG

**Sequence. S2: large-deletion dsDNA**

GCTGAGGGTGATGGGTTTGATAATGTCATGCCCTCACTCTTAGCACCCCACCCGGCGAGTCCTCACTGGGTGTAACACTAGCCTTCTGGAGGCAATCCTAGAGTCGGGCAGCAATTATTCCCACTTTTCAGAGACGGAGCAGGAAAGAGCCTGGGAGTTTGAAGGGCACCATACAGTTCAGTCCGTCAGGGACCATCTGGGAGAGCCTGTAACCTTCCTGTCCCGAAACTCTCAGTTTGCTCTCCTCTGCCATCATGCGAGGCTGCTGGCAGCGAGAACCGCAGCAACGCCTCAGCATGAAGGATGTGCACGCGCGTCTGCAGGCCCTGGCACAGGCGCCACCCAGTTACCTGGACGTTCTGGGCTAGGAGCCAAGTTTTGGTGCCAGGCCACCCTGGGCTCCCTCGGTGCCCAGGAGCTACCACATTCAAGTCTCTCACCCTCAGCATGTGG

**5 Supplementary References**

1. Brinkman EK, Chen T, Amendola M, van Steensel B. Easy quantitative assessment of genome editing by sequence trace decomposition. Nucleic Acids Res. 2014;42:e168.

2. Conant D, Hsiau T, Rossi N, Oki J, Maures T, Waite K, et al. Inference of CRISPR Edits from Sanger Trace Data. The CRISPR Journal. 2022;5:123–30.

3. Bloh K, Kanchana R, Bialk P, Banas K, Zhang Z, Yoo B-C, et al. Deconvolution of Complex DNA Repair (DECODR): Establishing a Novel Deconvolution Algorithm for Comprehensive Analysis of CRISPR-Edited Sanger Sequencing Data. CRISPR J. 2021;4:120–31.

4. Pedregosa F, Varoquaux G, Gramfort A, Michel V, Thirion B, Grisel O, et al. Scikit-learn: Machine Learning in Python. Journal of Machine Learning Research. 2011;12:2825–30.

5. Virtanen P, Gommers R, Oliphant TE, Haberland M, Reddy T, Cournapeau D, et al. SciPy 1.0: fundamental algorithms for scientific computing in Python. Nat Methods. 2020;17:261–72.
